# The stage‐specific roles of radiotherapy and chemotherapy in nodular lymphocyte predominant Hodgkin lymphoma patients: a propensity score‐matched analysis of the SEER database

**DOI:** 10.1002/cam4.3620

**Published:** 2020-11-28

**Authors:** Shijie Wang, Mingfang Jia, Jianglong Han, Rui Zhang, Kejie Huang, Yunfeng Qiao, Ping Chen, Zhenming Fu

**Affiliations:** ^1^ Cancer Center Renmin Hospital of Wuhan University Wuhan China; ^2^ Department of Health Management Renmin Hospital of Wuhan University Wuhan China

**Keywords:** chemotherapy, NLPHL, radiotherapy, stage‐specific

## Abstract

**Background:**

The stage‐specific roles of radiotherapy (RT) alone, chemotherapy alone, and combined RT and chemotherapy (CRT) for patients with nodular lymphocyte predominant Hodgkin lymphoma (NLPHL) have not been adequately evaluated.

**Methods:**

We analyzed patients with all stages of NLPHL enrolled in the Surveillance, Epidemiology, and End Results (SEER) registry from January 2000 to December 2015. Propensity score (PS) analysis with 1:1 matching (PSM) was performed to ensure the well‐balanced characteristics of the comparison groups. Kaplan–Meier and Cox proportional‐hazards models were used to evaluate the overall survival (OS), cancer‐specific survival (CSS), hazard ratios (HRs), and corresponding 95% confidence intervals (95% CI). Restricted mean survival times (RMST) were also used for the survival analyses.

**Results:**

For early‐stage patients, CRT was associated with the best survival, the mean OS was significantly improved by approximately 20 months (20 m), and the risk of death was reduced by more than 80%, both before and after PSM (*p* < 0.05). For advanced‐stage patients, none of RT alone, chemotherapy alone, or CRT had a significant effect on survival. Chemotherapy alone and CRT might be more beneficial for long‐term survival (RMST_120 m_: neither RT nor chemotherapy vs. chemotherapy alone vs. CRT = 104 m vs. 111 m vs. 108 m). Subgroup analysis of patients with early‐stage NLPHL showed that CRT was associated with better survival of elderly patients (improved OS = 43.8 m, HR = 0.14, *p* < 0.05). However, the survival benefits of treatments for young patients were not statistically significant. The efficacy of RT was significantly different between the age groups (*p*_for interaction_ = 0.020).

**Conclusions:**

These results from SEER data suggest that CRT may be considered for early‐stage NLPHL, especially for elderly patients. Further studies are needed to identify effective treatments in patients with advanced‐stage NLPHL.

## Graphical abstract

Combined radiotherapy and chemotherapy (CRT) is associated with the best survival of patients with the early‐stage nodular lymphocyte predominant Hodgkin lymphoma (NLPHL). Timely intervention is beneficial to survival of patients with NLPHL, especially for elderly patients in early stages. For advanced‐stage NLPHL, chemotherapy alone and CRT are likely to be associated with the long‐term survival benefits.

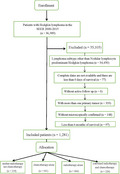

## 1 INTRODUCTION

Nodular lymphocyte predominant Hodgkin lymphoma (NLPHL) accounts for less than 10% of Hodgkin lymphomas (HLs).^1^ Its clinicopathological, immunological, and molecular features are different from those of classical HL (CHL). NLPHL develops slowly, tends to be inert, and has a better prognosis than CHL.^2^ Owing to its low incidence, few studies are defining the optimal treatment strategies for patients with NLPHL, especially for advanced‐stage patients.

Currently, treatment regimens for NLPHL are mostly based on experience in the treatment of CHL. Radiotherapy (RT), chemotherapy, and immunotherapy are all treatment options for NLPHL. Although the response rate to primary therapy exceeds 90%, late relapses are somewhat common (occurring in 10%–35% of the patients), which is different from CHL.^3^ Also, NLPHL can transform into a more aggressive lymphoma, as a result of treatment or progression of the disease, especially in patients who present with bulky disease, subdiaphragmatic disease, and splenic involvement.^4^ Approximately, 75% of the patients with NLPHL are diagnosed in the early stage (stage I and II), whereas patients with advanced‐stage disease (stage III and IV) are rare.^5^ In general, patients with early‐stage NLPHL are treated with local RT.^6^ However, the benefit of the addition of chemotherapy to RT alone in early‐stage disease remains controversial. Patients with advanced‐stage NLPHL usually receive chemotherapy, but the role of RT in advanced disease has not been determined.^6^

Nevertheless, RT and chemotherapy may both be valuable and currently applicable treatment options. A study of data from the National Cancer Database (NCDB) showed that RT improved survival in patients with all stages of NLPHL.^7^ However, no studies specifically addressed the relatively stage‐specific roles of RT alone, chemotherapy alone, and combined RT and chemotherapy (CRT) in NLPHL. Hence, we analyzed the data of patients with NLPHL from the Surveillance, Epidemiology, and End Results (SEER) registry by using conventional and propensity score matching (PSM) approaches.

## 2 PARTICIPANTS AND METHODS

### 2.1 Study population and data sources

SEER encompasses population‐based cancer registries covering approximately 28% of the U.S. population and records information on basic demographics, tumor site, histology type, stage, treatment, etc.^8^ SEER*stat software (version 8.3.4) was used to select patients from the SEER‐18 database who were diagnosed with HL in 2000–2015 (*n* = 36,389). Our study was limited to patients with NLPHL, using the lymphoma subtype recode/World Health Organization (WHO) 2008 classification (*n* = 1,939).^9^

**FIGURE 1:**
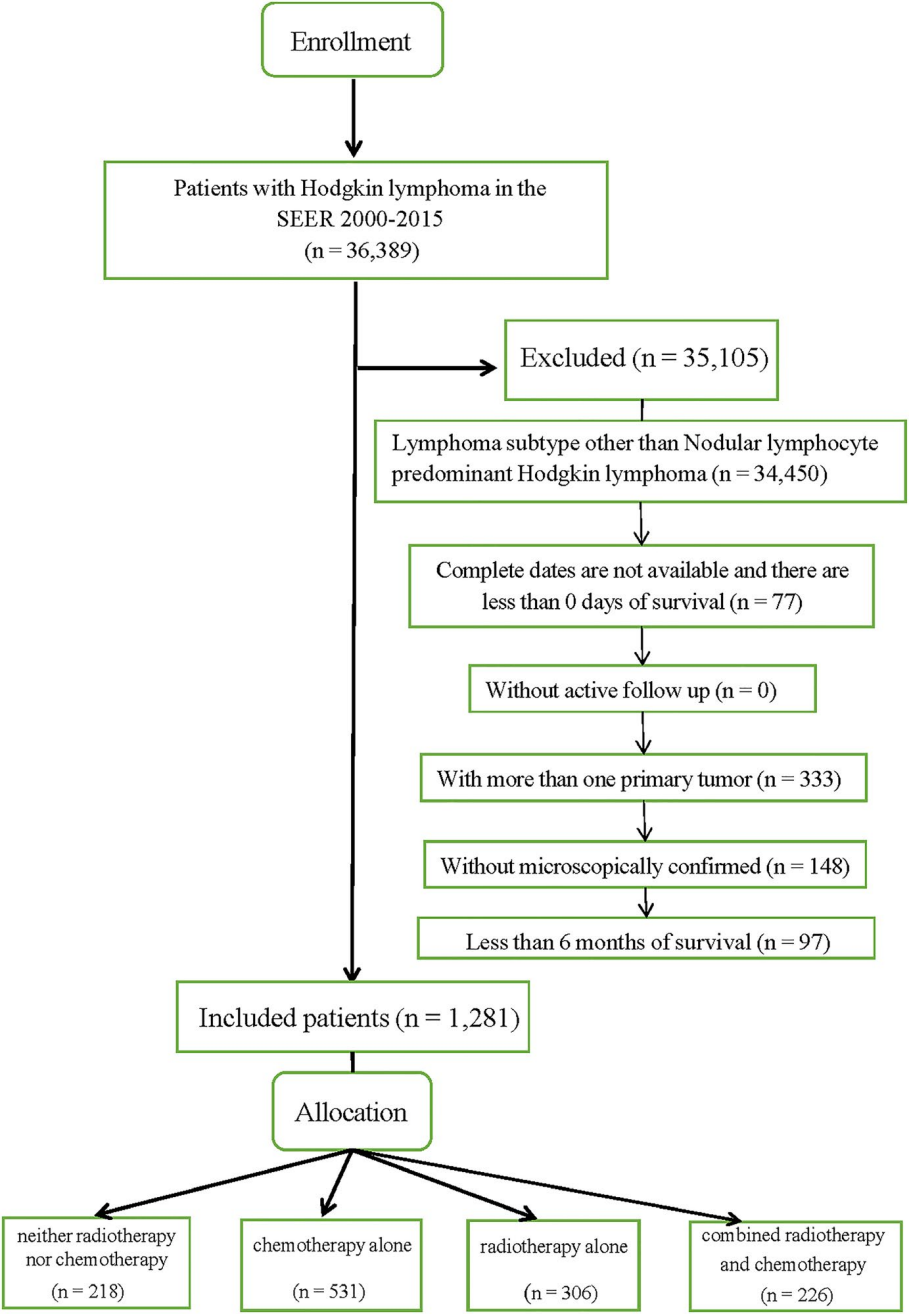
The flowchart of study population selection.

Included in our study were subjects with only one primary tumor, microscopic diagnosis confirmation, active follow‐up, and more than 6 months (6 m) of survival. Patients with unknown vital status were excluded. This resulted in a total of 1,281 patients in this study, including patients who received neither RT nor chemotherapy (*n* = 218, the control group), patients who received chemotherapy alone (*n* = 531, case group 1), patients who received RT alone (*n* = 306, case group 2), and patients who received CRT (*n* = 226, case group 3) (Figure 1). Pertinent demographics and treatment characteristics were included in this analysis. Treatment‐related variables coded in SEER reflect initial treatment only. No specific chemotherapy protocol could be ascertained. We categorized patients into older (≥45 years old) and younger patients (<45 years old) according to the stratification of age recommended by the WHO.^10^ Lower family income was defined as those with less than 5000 dollars per annum.

### 2.2 PSM

PSM is a tool to reduce the selection bias in nonrandomized studies. PSM can adjust potentially confounding factors to improve the balance between groups and enhance comparability.^11^ This makes the results more credible. A multivariate logistic regression model was used to calculate propensity scores for each patient. Propensity 1:1 nearest neighbor matching, which matched patients treated with chemotherapy and those treated without chemotherapy, was employed (PSM model 1). Similarly, PSM was used to match patients treated with RT and those treated without RT (PSM model 2). The covariate balance was examined by the standardized deviation. Covariates were selected by the same strategy as shown in the following section.

### 2.3 Statistical analysis

Categorical data were described with absolute frequency counts and percentages; continuous data were described by means (since the median survival had not yet been reached as of the date of analysis). General linear models and Mantel–Haenszel chi‐square tests were used to compare the distribution of demographic characteristics. The primary objective was to evaluate overall survival (OS) and cause‐specific survival (CSS).

The statistical analyses consisted of multiple steps. First, Kaplan–Meier curves were used to assess the association of variables with survival. Subsequently, significant variables in the univariate analysis were entered into the multivariate Cox regression models. Variables that remained significant were used for the final Cox regression analysis and were selected to generate the propensity scores. The restricted mean is a measure of average survival from time 0 to a specified time point and may be estimated as the area under the survival curve up to that point.^12^ Restricted mean survival times (RMSTs), which were calculated by the package “survRM2” of the R Project,^13^ were used to assess the survival of patients in advanced stages. The data from each treatment group were insufficient for the Cox regression analysis. The truncation time points for the calculation of RMSTs were 36 m, 60 m, and 120 m. The survival analyses above were conducted in the original data set and the matched data sets (PSM model 1 and PSM model 2). Finally, we conducted subgroup analyses according to age group and the presence of B symptoms.

Any causes of death or the survival status of patients were censored at the time of death or the last follow‐up. Age was used as the timescale for all models, with the entry time defined as age at diagnosis and exit time defined as age at death, last follow‐up, or 31 December 2015, whichever came first. A *p*‐value of ≤0.05 (two‐sided probability) was considered statistically significant. All analyses were conducted using SPSS 23 (IBM Corp, Armonk, NY, USA) and R version 3.6.1.

## 3 RESULTS

### 3.1 Baseline characteristics

The distributions of patient characteristics are presented in Table 1 for the study groups categorized by receipt of RT, and receipt of chemotherapy. A total of 1281 patients were selected in the study, including patients in stage I (41.1%), stage II (28.1%), stage III (20.1%), stage IV (6.6%), and unknown stage (4.0%). The mean age of the patients was 38 years old (range, 2–88). The majority of the patients were male (69.6%) and white (68.8%), and lived in Pacific Coast/Alaska (41.4%) and East (41.4%) regions. The proportion of patients that had B symptoms (12.6%) was lower than that of patients who did not have them (61.0%). Patients who were younger, had stage II or above disease, had B symptoms, and who did not receive RT or surgery, were more likely to receive chemotherapy. Patients who had early‐stage disease, had B symptoms, did not receive chemotherapy, and who received surgery, were more likely to receive RT. In early‐stage NLPHL, the proportion of patients who received RT was slightly higher than those who did not receive RT (stage I, 56.0% vs. 44.0%; stage II, 51.7% vs. 48.3%). After PSM, the distributions of most demographic and clinical factors were well balanced between groups.

**TABLE 1:** Selected baseline characteristics of patients with NLPHL, before and after PSM, SEER 2000–2015

Variable	Before PSM							PSM model 1^1^				PSM model 2^2^			
	Total *N*	No‐CT^3^	CT	*p* ^4^	No‐RT^3^	RT	*p* ^4^	Total *N*	No‐CT^3^	CT	*p* ^4^	Total *N*	No‐RT^3^	RT	*p* ^4^
		(%)	(%)		(%)	(%)			(%)	(%)			(%)	(%)	
*Age*															
Years, mean (SD)	1281	41.7 (16.9)	36.6 (19.2)	<0.001	37.8 (19.6)	39.9 (16.6)	0.037	814	39.8 (16.5)	39.9 (18.6)	0.997	762	38.7 (19.6)	40.4 (16.7)	0.213
<45 (%)	785	37.3	62.7	<0.001	59.4	40.6	0.414	482	50.8	49.2	0.568	452	51.3	48.7	0.376
≥45 (%)	496	46.6	53.4		57.1	42.9		332	48.8	51.2		310	48.1	51.9	
*Gender (%)*				0.85			0.905				0.449				0.757
Male	891	41.1	58.9		58.4	41.6		560	50.9	49.1		516	49.6	50.4	
Female	390	40.5	59.5		58.7	41.3		254	48	52		246	50.8	49.2	
*Race (%)*				0.203			0.531				0.13				0.641
White	881	39.7	60.3		57.9	42.1		560	48.2	51.8		518	50.6	49.4	
Others	400	43.5	56.5		59.8	40.3		254	53.9	46.1		244	48.8	51.2	
*Marital status (%)*				<0.001			0.001				0.001				0.002
Married	570	41.9	58.1		53.2	46.8		390	45.6	54.4		356	43.5	56.5	
Unmarried	635	37.3	62.7		62	38		369	51.2	48.8		353	54.4	45.6	
Unknown	76	63.2	36.8		68.4	31.6		55	72.7	27.3		53	64.2	7	
*CHADA Region (%)*				0.205			0.035				0.028				0.01
Pacific Coast/Alaska	531	40.3	59.7		61.4	38.6		329	49.5	50.5		297	57.6	42.4	
East	531	41.4	58.6		58.6	41.4		327	47.1	52.9		331	44.4	55.6	
Northern Plains	148	45.9	54.1		48	52		108	63	37		96	46.9	53.1	
Southwest	71	31	69		57.7	42.3		50	44	56		38	47.4	52.6	
*Income (%)*				0.873			0.425				0.887				0.372
<5000	532	41.2	58.8		59.8	40.2		334	50.3	49.7		296	48	52	
≥5000	749	40.7	59.3		57.5	42.5		480	49.8	50.2		466	51.3	48.7	
*Lymphoma stage (%)*				<0.001			<0.001				<0.001				0.177
Stage I	527	59.6	40.4		44	56		377	57.6	42.4		399	49.9	50.1	
Stage II	360	33.9	66.1		48.3	51.7		229	44.5	55.5		240	45.8	54.2	
Stage III	257	16.3	83.7		88.3	11.7		129	32.6	67.4		75	60	40	
Stage IV	85	9.4	90.6		84.7	15.3		31	25.8	74.2		26	50	50	
Unknown	52	73.1	26.9		84.6	15.4		48	79.2	20.8		22	63.6	36.4	
*B symptoms (%)*				<0.001			<0.001				<0.001				0.004
No	781	41.5	58.5		54.3	45.7		483	49.3	50.7		447	45	55	
Yes	162	24.1	75.9		69.1	30.9		88	33	67		88	59.1	40.9	
Unknown	338	47.6	52.4		63	37		243	57.6	42.4		227	56.4	43.6	
*Extra nodal involvement (%)*				0.37			0.124				0.634				0.825
No	1248	40.7	59.3		58.8	41.2		796	49.9	50.1		741	50.1	49.9	
Yes	33	48.5	51.5		45.5	54.5		18	55.6	44.4		21	47.6	52.4	
*Chemotherapy (%)*				‐			<0.001				‐				0.041
No	524	‐	‐		41.6	58.4		407	‐	‐		340	54.1	45.9	
Yes	757	‐	‐		70.1	29.9		407	‐	‐		422	46.7	53.3	
*Radiotherapy (%)*				<0.001							0.4				‐
No	749	29.1	70.9		‐	‐		424	51.4	48.6		381	‐	‐	
Yes	532	57.5	42.5		‐	‐		390	48.5	51.5		381	‐	‐	
*Surgery (%)*				0.055			0.037				0.62				0.554
No	569	38	62		61.7	38.3		351	49	51		304	51.3	48.7	
Yes	712	43.3	56.7		55.9	44.1		463	50.8	49.2		458	49.1	50.9	

Abbreviations: CT, chemotherapy; *N*, number of cases; NLPHL, nodular lymphocyte predominant Hodgkin lymphoma; PSM, propensity score matching; RT, radiotherapy; SEER, Surveillance, Epidemiology, and End Results.

Selected covariates for propensity score analysis with 1:1 matching were: age, region, stage, and chemotherapy/radiotherapy.

^1^ PSM was performed to match patients treated with chemotherapy and those treated without chemotherapy.

^2^ PSM was performed to match patients treated with radiotherapy and those treated without radiotherapy.

^3^ “No‐CT” means patients who did not receive chemotherapy. “No‐RT” means patients who did not receive radiotherapy.

^4^ *p*‐values were derived from rank test for continuous variables and X^2^ test for categorical variables.

### 3.2 The role of chemotherapy and RT in early‐stage NLPHL

Table 2 shows the survival of patients with early‐stage NLPHL in different treatment groups, before and after PSM (more details about stage I and stage II separately are shown in Tables S1 and S2). The group of patients who received neither chemotherapy nor RT was used as the reference in the following comparisons. For patients with early‐stage NLPHL, CRT was associated with the best OS (before PSM: improved OS = 19.5 m, *p* = 0.001; HR_,_ 95% CI = 0.19, 0.05–0.69, *p = *0.011) (after PSM: PSM model 1: improved OS = 19.4 m, *p* = 0.001; HR, 95% CI = 0.12, 0.03–0.46, *p = *0.002. PSM model 2: improved OS = 20.6 m, *p* = 0.001; HR, 95% CI = 0.11, 0.03–0.49, *p = *0.001). The 10‐year CSS was nearly 100% for patients who received CRT. RT alone was also found to be associated with an improved OS of approximately 15 m (*p* > 0.05), improved the CSS by around 10–12 m (before PSM, after PSM: *p = *0.034, 0.106, respectively), and decreased the HR by around 60%–80% (before PSM, after PSM: *p_OS_*_ = _0.022, 0.003; *p_CSS_*_ = _0.036, 0.109, respectively). Although chemotherapy alone had certain survival benefits (before PSM, after PSM: improved OS = 12.1 m, 9.2 m; improved CSS = 5.3 m, 4.7 m, respectively), most of these increases were not statistically significant.

**TABLE 2:** Multivariate analysis of OS and CSS in patients with early‐stage NLPHL, before and after PSM, SEER 2000–2015

Therapy	*N*	OS				CSS			
		10y OS (%)	Mean (95% CI)	HR (95% CI)	*p* ^1^	10y CSS (%)	Mean (95% CI)	HR (95% CI)	*p* ^1^
*Before PSM*									
Neither RT nor CT (as ref.)	143	87.7	166.4 (157.3–175.5)	1		95	176.2 (170.3–182.1)	1	
CT alone	263	94.7	178.5 (173.9–183.0)	0.48 (0.21–1.11)	0.086	96.9	181.5 (177.9–185.1)	0.67 (0.20–2.20)	0.506
RT alone	293	93.5	181.6 (176.7–186.5)	0.38 (0.17–0.87)	0.022	98.9	188.5 (186.4–190.6)*	0.17 (0.03–0.89)	0.036
CRT	188	98.6	185.9 (182.4–189.3)*	0.19 (0.05–0.69)	0.011	100	^2^/	/	/
*After PSM*									
^3^PSM Model 1									
Neither RT nor CT (as ref.)	143	87.7	166.4 (157.3–175.5)	1		95	176.2 (170.1–182.1)	1	
CT alone	146	93	175.6 (168.6–182.6)	0.30 (0.11–0.80)	0.017	98.9	180.9 (176.0–185.8)	0.18 (0.02–1.55)	0.119
CRT	177	98.6	185.8 (182.2–189.3)*	0.12 (0.03–0.46)	0.002	100	/	/	/
^4^PSM Model 2									
Neither RT nor CT (as ref.)	133	81.7	165.3 (155.8–174.9)	1		94.6	175.6 (169.3–182.0)	1	
RT alone	133	94.6	180.4 (173.9–186.8)	0.15 (0.04–0.53)	0.003	98.3	185.1 (181.5–188.8)	0.17 (0.02–1.48)	0.109
CRT	188	98.6	185.9 (182.4–189.3)*	0.11 (0.03–0.40)	0.001	100	/	/	/

Abbreviations: 10y CSS, CSS rate at the tenth years; 10y OS, OS rate at the tenth years; CI, confidence interval; CRT, combined radiotherapy and chemotherapy; CSS, cancer‐specific survival; CT, chemotherapy; HR, hazard ratio; *N*, number of cases; NLPHL, Nodular lymphocyte predominant Hodgkin lymphoma; OS, overall survival; PSM, propensity score matching; ref, reference; RT, radiotherapy; SEER, Surveillance, Epidemiology, and End Results.

^1^ *p*‐values were derived from final multivariate Cox proportional‐hazards models.

^2^ “/” means no patient reached an end event.

^3^ PSM model 1: PSM was performed to match patients treated with chemotherapy and those treated without chemotherapy.

^4^ PSM model 2: PSM was performed to match patients treated with radiotherapy and those treated without radiotherapy.

* *p*‐value which was derived from Kaplan–Meier survival analysis was <0.05.

### 3.3 The role of chemotherapy and RT in advanced‐stage NLPHL

Table 3 presents the survival of patients with advanced‐stage NLPHL in different treatment groups, before and after PSM (more details are shown in Tables S1–S3). Most patients with advanced‐stage NLPHL received chemotherapy alone (74.9%), and few of them received RT alone (2.0%). Taking the group of patients who received neither chemotherapy nor RT as the reference, none of the treatments provided statistically significant improvements in the survival of patients. The results were consistent before PSM and after PSM. During early periods (36 m), no treatment group showed better survival. With the extension of follow‐up, the survival benefits from chemotherapy alone and CRT seemed to increase over time (control group vs. case group 1 vs. case group 3; RMST_36 m_ = 34.8 m vs. 34.5 m vs. 34.0 m; RMST_60 m_ = 56.1 m vs. 56.2 m vs. 55.1 m; RMST_120 m_ = 104.7 m vs. 106.3 m vs. 108.1 m).

**TABLE 3:** RMST of patients with advanced‐stage NLPHL, before and after PSM, SEER 2000–2015

Therapy	*N*	RMST‐OS (months)						RMST‐CSS (months)					
		36 months	*p* ^1^	60 months	*p* ^1^	120 months	*p* ^1^	36 months	*p* ^1^	60 months	*p* ^1^	120 months	*p* ^1^
		(95% CI)		(95% CI)		(95% CI)		(95% CI)		(95% CI)		(95% CI)	
*Before PSM*													
Neither RT nor CT (as ref.)	43	34.8 (33.5–36.2)		56.1 (52.7–59.5)		104.7 (94.2–115.1)		35.1 (33.8–36.4)		57.4 (54.4–60.4)		110.5 (101.6–119.3)	
CT alone	256	34.5 (33.7–35.2)	0.642	56.2 (54.6–57.8)	0.959	106.3 (101.9–110.7)	0.781	34.9 (34.3–35.6)	0.827	57.4 (56.1–58.7)	0.997	111.9 (108.3–115.5)	0.772
CRT	36	34.0 (31.9–36.1)	0.499	55.1 (50.6–59.7)	0.74	108.1 (97.1–119.1)	0.657	34.8 (33.3–36.2)	0.742	56.6 (52.8–60.3)	0.725	111.0 (101.3–120.7)	0.934
*After PSM*													
^2^PSM model 1													
Neither RT nor CT (as ref.)	43	34.8 (33.5–36.2)		56.1 (52.7–59.5)		104.7 (94.2–115.1)		35.1 (33.8–36.4)		57.4 (54.4–60.4)		110.5 (101.6–119.3)	
CT alone	45	35.7 (35.3–36.1)	0.228	58.2 (55.9–60.6)	0.317	111.4 (103.4–119.5)	0.313	35.8 (35.5–36.2)	0.276	59.1 (57.4–60.8)	0.332	117.4 (112.2–122.5)	0.187
^3^PSM model 2													
Neither RT nor CT (as ref.)	37	34.9 (33.5–36.4)		56.5 (53.0–60.1)		104.9 (93.8–116.0)		34.9 (33.5–36.4)		57.0 (53.6–60.5)		108.9 (98.7–119.1)	
CRT	36	34.0 (33.5–36.4)	0.457	55.1 (50.6–59.7)	0.64	108.1 (97.1–119.1)	0.685	34.8 (33.3–36.2)	0.868	56.6 (52.8–60.3)	0.86	111.0 (101.3–120.7)	0.772

Abbreviations: CI, confidence interval; CRT, combined radiotherapy and chemotherapy; CT, chemotherapy; *N*, number of cases; NLPHL, Nodular lymphocyte predominant Hodgkin lymphoma; PSM, propensity score matching; ref, reference; RMST, restricted mean survival times; RT, radiotherapy; SEER, Surveillance, Epidemiology, and End Results.

^1^ *p*‐values were derived from the RMST‐based analyses.

^2^ PSM model 1: PSM was performed to match patients treated with chemotherapy and those treated without chemotherapy.

^3^ PSM model 2: PSM was performed to match patients treated with radiotherapy and those treated without radiotherapy.

Subgroup analyses.

Table 4 shows the subgroup analyses of OS, according to age group and the presence of B symptoms, before and after PSM. For younger patients (<45 years old) with early‐stage NLPHL, chemotherapy and RT did not bring statistically significant survival benefits. Of the 38 younger patients in the advanced stage, 23 patients received RT or chemotherapy, and had not died at the time of follow‐up. For older patients (≧45 years old) with early‐stage NLPHL, RT alone was associated with better survival (improved OS = 43.4 m, *p* = 0.062), and CRT significantly improved the survival (improved OS = 43.8 m, *p* = 0.002). After PSM, both RT alone and CRT were significantly associated with reduced HR (*p* < 0.05). The role of RT alone in patients with early‐stage NLPHL was significantly different between young and elderly patients (*p*_for interaction_ = 0.020). For patients without B symptoms, the survival benefits, which were associated with RT and chemotherapy, were not statistically significant both before and after PSM (*p* > 0.05). The number of patients with B symptoms was too small to compare survival among the treatments.

**TABLE 4:** Final multivariate analysis of OS by different subgroups in patients with NLPHL, SEER 2000–2015

Therapy	Before PSM			After PSM^1^	
	*N*	Mean survival (95% CI)	*p* ^2^	HR (95% CI)	*p* ^3^
*Age <45, early stage*					
Neither RT nor CT (as ref.)	87	178.4 (172.6–184.1)		1	
RT alone	183	185.4 (181.7–189.0)	0.403	1.08 (0.16–7.06)^5^	0.94
CRT	124	187.6 (184.9–190.3)	0.143	0.19 (0.02–1.87)^6^	0.156
*Age ≧ 45, early stage*					
Neither RT nor CT (as ref.)	56	130.6 (114.1–147.1)		1	
CT alone	80	154.5 (142.0–167.0)	0.441	0.77 (0.29–2.04)^4^	0.605
RT alone	132	174.0 (163.7–184.2)	0.062	0.08 (0.01–0.57)^5^	0.011
CRT	64	174.4 (167.2–181.6)	0.002	0.14 (0.03–0.66)^6^	0.013
*Without B symptom*					
Early stage (IA, IIA)					
Neither RT nor CT (as ref.)	79	135.5 (129.4–141.6)		1	
CT alone	155	139.0 (135.1–142.9)	0.646	0.77 (0.15–3.96)^4^	0.758
RT alone	208	136.1 (132.6–139.7)	0.923	0.45 (0.08–2.71)^5^	0.385
CRT	119	141.9 (139.7–144.1)	0.119	0.19 (0.02–1.86)^6^	0.155
Advanced stage (IIIA, IVA)					
Neither RT nor CT (as ref.)	23	115.5 (94.9–136.1)		1	
CT alone	159	130.2 (123.5–136.8)	0.211	0.26 (0.03–2.29)^4^	0.223
CRT	23	123.7 (105.5–141.8)	0.675	0.82 (0.17–4.08)^6^	0.809

Abbreviations: CI, confidence interval; CRT, combined radiotherapy and chemotherapy; CT, chemotherapy; NLPHL, Nodular lymphocyte predominant Hodgkin lymphoma; OS, overall survival; PSM, propensity score matching; ref., reference; RT, radiotherapy; SEER, Surveillance, Epidemiology, and End Results.

Groups with no valid results are not shown in the table.

^1^ Selected covariates for Propensity score (PS) analysis with 1:1 matching were different at different stages.

^2^ *p*‐values were derived from Kaplan–Meier survival analysis.

^3^ *p*‐values were derived from the final multivariate Cox proportional‐hazards models that insignificant variables were dropped.

^4^ PSM model 1: PSM was performed to match patients treated with chemotherapy and those treated without chemotherapy.

^5^ PSM model 2: PSM was performed to match patients treated with radiotherapy and those treated without radiotherapy.

^6^ Evaluating the association of CRT with survival in PSM model 1 or 2, respectively. The better results were showed here.

## 4 DISCUSSION

To our knowledge, the present study is the first SEER analysis using PSM to assess the stage‐specific roles of RT alone, chemotherapy alone, and CRT in the treatment of NLPHL. The stage‐specific survival benefits seen from the large SEER database suggested that the use of RT and chemotherapy in the management of NLPHL should be stage‐specific.

Owing to the low incidence of NLPHL, the number of prospective randomized controlled trials (RCTs) conducted to evaluate the optimum treatment for all stages has been limited so far. For early‐stage NLPHL, RT alone has generally been considered to be able to control disease and achieve a high survival rate.^14^, ^15^, ^16^, ^17^, ^18^ However, with widespread concern about the long‐term risk of RT, the use of chemotherapy alone has been further studied. Some studies have reported that lymphomas treated with chemotherapy alone were likely to relapse after treatment, and that chemotherapy alone could not adequately control the disease.^14^, ^19^, ^20^ On the contrary, the controversy over whether or not chemotherapy should be added to RT remained. Also, some studies have suggested that combination chemotherapy was ineffective,^14^, ^15^, ^17^, ^19^ while others found that the addition of chemotherapy could reduce the long‐term complications of RT.^21^, ^22^, ^23^ An RCT indicated that chemotherapy combined with reduced‐dose RT was equally effective and less toxic than RT alone^23^ Our research reinforced that RT played an important role in the treatment of early‐stage NLPHL, and found that CRT was associated with the best survival. Independently, compared to RT alone, CRT could reduce patients’ death risk by more than half; however, this decrease was not statistically significant. Optimizing the risk/benefit ratio of treatment by minimizing toxicity is the primary objective of most clinical studies on early‐stage NLPHL. Thus, reducing the dose of RT by combining it with chemotherapy, which can achieve the same efficacy with reduced toxicity, might be the optimal option for early‐stage patients.^5^, ^24^ In line with our findings, the recent HD7 to HD15 trials of the German Hodgkin Study Group (GHSG) indicated that the first‐line treatment for early‐stage NLPHL should be local RT after short‐term chemotherapy.^25^

The optimal therapeutic strategy for the advanced‐stage NLPHL has yet to be defined. Currently, evidence for the treatment of advanced‐stage NLPHL mainly comes from retrospective analyses of CHL and single‐institution published experiences.^26^ The GHSG in 2008 reported that the complete remission rate and freedom from treatment failure rate were similar for advanced‐stage NLPHL and CHL.^27^ Thus, patients with advanced‐stage NLPHL were generally treated the same way as patients with CHL. Chemotherapy was recommended for patients with advanced‐stage NLPHL, and the addition of RT was optional.^6^ In the absence of RCTs, controversies exist regarding the best chemotherapy regimen.^28^, ^29^ National Comprehensive Cancer Network guidelines suggest that the chemotherapy regimens of either CHL or B‐cell non‐Hodgkin lymphoma are options for NLPHL.^6^ To the best of our knowledge, phase III RCTs studying the role of RT in treating advanced‐stage NLPHL are not available, except for one retrospective analysis based on the NCDB database.^7^ The NCDB study concluded that RT was associated with prolonged OS in all stages of NLPHL (median OS for advanced‐stage: 54.1 vs. 39.6 months, *p* = 0.06). However, our analysis did not find that RT alone, chemotherapy alone, and CRT significantly prolonged the survival of patients with advanced‐stage NLPHL. Although chemotherapy alone and CRT seemed to have benefits for long‐term survival, the sample size might not have been large enough; besides, NLPHL patients generally have long survival. Thus, these factors might result in inadequate power in the statistical analysis.

Being older than 45 years old was generally considered to be a poor prognostic factor.^18^ We found that CRT increased survival by 43.8 m for older patients but only 9.2 m for younger patients. Particularly, we found that elderly patients could benefit more from RT than younger patients, and the difference was statistically significant (*p*_for interaction_ = 0.020). This may be due to the commonly longer survival of younger patients. Additionally, this indicated that elderly patients needed more intensive treatment because of their poor prognosis. In general, our results suggested that early‐stage patients over 45 years old might benefit from timely treatments, of which CRT might be the appropriate option. For younger patients, CRT was also associated with better survival than RT alone.

B symptoms are rare in patients with NLPHL, and only 12.6% of the patients in this study suffered from these symptoms. Consistent with previous findings,^30^, ^31^ we found that the presence of B symptoms was associated with worse survival (OS, 126.3 m vs. 136.3 m). Current studies suggest that patients with stage IA NLPHL are at low risk, and combined therapy is not recommended for this group of patients.^16^, ^18^ Similarly, in the subgroup analysis of early‐stage patients without B symptoms, we did not find significant survival benefits from RT or chemotherapy, suggesting that additional therapy might be unnecessary for low‐risk patients. Due to limitations of our data, we were not able to compare the survival benefits between different treatments in patients with B symptoms, as well as in patients with other poor prognostic factors, such as bulky disease and splenic involvement.

Death in NLPHL patients is often caused by late treatment‐related effects, such as secondary malignant tumors, rather than lymphoma‐related complications.^25^ Considering the indolent course of NLPHL, many patients might be managed by watchful waiting.^32^ However, just watching and waiting might lead to unfavorable development or transformation into a more aggressive type, resulting in a diminished potential for cure.^33^ A retrospective study found that early treatment with chemotherapy or RT significantly reduced the risk of progression compared to watchful waiting.^34^ Our findings also demonstrated the effectiveness of chemotherapy and RT in early‐stage patients. Therefore, prompt intervention with curative intent might be a reasonable choice for appropriate patients in the early stages.

Unlike RCT data, the data from the SEER registry are usually very complete and represent the real‐world patient population. Nevertheless, we acknowledge several limitations of this study. First, the possibility of bias is a concern. The participants in the study were recruited through a representative national database, which reduced possible selection bias. We excluded patients who survived less than 6 months to reduce immortal time bias.^35^ PSM was used to reduce the bias caused by the imbalanced distribution of measured covariates. The results remained stable before and after PSM. Second, the study was based on registry data, which contained limited information; for example, the data lacked information on treatment regimens and potential risk factors. Although it would be preferable to obtain more details, this study aimed to describe the overall role of RT alone, chemotherapy alone, and CRT in all stages of NLPHL. In this regard, we believe that the available data can serve the aim well. Moreover, the role of rituximab cannot be ignored.^36^, ^37^, ^38^ However, our results also suggested that rituximab alone could not fully control the disease. We analyzed the data of NLPHL patients enrolled in SEER before 2000, which were collected before the rituximab era (Table S4). The results were essentially identical. Therefore, RT and chemotherapy are still valuable options for the treatment of NLPHL. Finally, although the SEER‐Medicare database reports comorbidity, detailed radiation, and second‐line chemotherapy information, patients in the database are limited to an older population (>65 years of age), which is not relevant to NLPHL.

Given the rarity of NLPHL, prospective clinical trials with enough power are not easily conducted, especially for specific subgroups. Although some limitations existed, this study made use of the largest database and provided valuable evidence with considerable significance in informing the treatment paradigms for NLPHL patients.

## 5 CONCLUSION

In conclusion, our “real‐word” analysis of SEER data did not produce results that substantially differ from those of current studies, and rather confirmed previous results. For early‐stage patients, we found that CRT was associated with the best survival. Especially for elderly patients in the early stages, a timely intervention was beneficial to survival. For advanced‐stage patients, chemotherapy alone and CRT were likely to be associated with long‐term survival benefits, but these associations were not statistically significant. More effective treatment strategies and analyses of event‐free survival for patients with NLPHL remain to be explored in phase III RCTs.

## CONFLICT OF INTEREST

The authors have no conflicts of interest.

## AUTHOR CONTRIBUTIONS

Zhenming Fu and Mingfang Jia designed and directed the study. Shijie Wang analyzed the data. Jianglong Han and Yunfeng Qiao helped with the statistical analysis and data cleaning. Shijie Wang, Rui Zhang, and Zhenming Fu drafted the manuscript. Kejie Huang, Mingfang Jia and Ping Chen provided clinical insights, did the literature review and helped with the drafting of the manuscript. All authors reviewed and approved the final draft. This article is not under consideration elsewhere.

## Supporting information

Supplementary Material

## Data Availability

The data that support the findings of this study are openly available in the Surveillance, Epidemiology, and End Results (SEER) database.
