# Supplementary material for: The stage‐specific roles of radiotherapy and chemotherapy in nodular lymphocyte predominant Hodgkin lymphoma patients: a propensity score‐matched analysis of the SEER database
Source: Cancer Med. 2020 Nov 28;10(2):540–51. doi: 10.1002/cam4.3620 (PMC7877359; doi:10.1002/cam4.3620)
Supplement: Supplementary file 1 — Supplementary Material [file CAM4-10-540-s001.docx]

| Supplementary table 1. Univariate and multivariate analysis of OS and CSS in patients with stage I-IV NLPHL, respectively, before PSM, SEER 2000-2015. | | | | | | | | | | | | |
| --- | --- | --- | --- | --- | --- | --- | --- | --- | --- | --- | --- | --- |
|  | N | OS (months) | | | | |  | CSS (months) | | | | |
|  |  | 10y OS (%) | Mean (95% CI) | *P*^1^ | HR(95%CI) | *P^2^* |  | 10y CSS (%) | Mean (95% CI) | *P*^1^ | HR(95%CI) | *P^2^* |
| **All stage** | | | | | | | | | | | | |
| **Radiotherapy**  No  Yes | 749  532 | 87.4  95.4 | 169.0 (164.8-173.3)  183.0 (179.9-186.2) | < 0.001 | 1.00  0.54 (0.29-0.99) | 0.046 |  | 94.0  98.8 | 178.1 (175,2-181.1)  188.1 (186.5-189.8) | < 0.001 | 1.00  0.36 (0.13-1.01) | 0.053 |
| **Chemotherapy**  No  Yes | 524  757 | 91.8  91.1 | 176.6 (172.1-181.2)  175.3 (171.6-179.0) | 0.616 | 1.00  0.67 (0.41-1.09) | 0.108 |  | 97.0  95.4 | 185.6 (183.0-188.2)  181.9 (179.3-184.5) | 0.132 | 1.00  0.87 (0.41-1.84) | 0.708 |
| **Therapy**  Neither RT nor CT  CT alone  RT alone  CRT | 218  531  306  226 | 85.8  88.2  93.8  96.9 | 168.0 (160.2-175.8)  169.1 (164.2-173.9)  182.0 (177.2-186.7)  183.2 (178.9-187.4) | 0.924  0.018  0.004 | 1.00  0.87 (0.51 - 1.49)  0.46 (0.21 - 1.00)  0.45 (0.19 - 1.09) | 0.610  0.049  0.076 |  | 94.4  93.8  98.9  98.5 | 178.9 (173.7-184.1)  176.9 (173.3-180.5)  188.6 (186.5-190.6)  186.6 (183.8-189.3) | 0.689  0.009  0.058 | 1.00  1.09 (0.50 - 2.39)  0.21 (0.04 - 1.04)  0.49 (0.13 - 1.90) | 0.825  0.056  0.304 |
| **Stage I** | | | | | | | | | | | | |
| **Radiotherapy**  No  Yes | 232  295 | 93.2  96.0 | 175.5 (169.7-181.2)  184.5 (180.7-188.3) | 0.136 | 1.00  0.35 (0.14-0.87) | 0.024 |  | 96.8  99.3 | 181.1 (177.3-185.0)  189.2 (187.7-190.7) | 0.027 | 1.00  0.10 (0.01-0.83) | 0.033 |
| **Chemotherapy**  No  Yes | 314  213 | 92.0  98.3 | 177.9 (172.3-183.5)  186.4 (183.4-189.3) | 0.011 | 1.00  0.22 (0.06-0.77) | 0.018 |  | 97.6  99.0 | 186.6 (183.7-189.6)  187.2 (184.8-189.7) | 0.472 | 1.00  0.40 (0.08-2.14) | 0.287 |
| **Therapy**  Neither RT nor CT  CT alone  RT alone  CRT | 110  122  204  91 | 87.9  98.3  94.7  98.4 | 166.3 (156.4-176.2)  183.0 (178.8-187.1)  182.6 (177.2-188.0)  187.1 (183.5-190.8) | 0.016  0.060  0.006 | 1.00  0.18 (0.04-0.84)  0.32 (0.12-0.86)  0.11 (0.01-0.88) | 0.029  0.023  0.038 |  | 95.3  98.3  98.9  100.0 | 176.3 (169.9-182.8)  183.0 (178.8-187.1)  188.7 (186.2-191.2)  / | 0.361  0.046  0.058 | 1.00  0.44 (0.08-2.45)  0.11 (0.013-1.02)  - | 0.351  0.052  - |
| **Stage II** | | | | | | | | | | | | |
| **Radiotherapy**  No  Yes | 174  186 | 91.0  95.3 | 170.2 (163.2-177.2)  177.5 (171.7-183.3) | 0.175 | 1.00  0.25 (0.07-0.90) | 0.033 |  | 95.2  99.5 | 175.9 (170.6-181.2)  184.0 (182.2-185.9) | 0.072 | 1.00  0.13 (0.01-1.38) | 0.090 |
| **Chemotherapy**  No  Yes | 122  238 | 89.1  94.6 | 170.1 (160.5-179.8)  176.1 (170.7-181.5) | 0.559 | 1.00  0.41 (0.11-1.52) | 0.184 |  | 97.6  97.5 | 178.7 (174.1-183.3)  181.6 (178.3-184.9) | 0.954 | 1.00  0.47 (0.07-3.41) | 0.458 |
| **Therapy**  Neither RT nor CT  CT alone  RT alone  CRT | 33  141  89  97 | 89.3  91.4  89.7  98.9 | 168.2 (150.1-186.3)  168.0 (160.6-175.3)  164.1 (154.3-173.8)  181.2 (176.3-186.1) | 0.801  0.558  0.169 | 1.00  0.51 (0.09-2.92)  0.31 (0.05-1.99)  0.10 (0.01-1.11) | 0.505  0.312  0.096 |  | 93.8  98.9  95.6  100.0 | 174.4 (160.1-188.8)  173.4 (168.0-178.8)  172.1 (168.4-175.8)  / | 0.862  0.440  0.046 | 1.00  0.69 (0.06-7.65)  0.20 (0.01-3.59)  - | 0.693  0.198  - |
| **Stage III** | | | | | | | | | | | | |
| **Radiotherapy**  No  Yes | 227  30 | 79.1  88.9 | 159.2 (149.2-169.2)  148.8 (131.5-166.1) | 0.745 | 1.00  1.36 (0.40-4.69) | 0.623 |  | 90.6  91.9 | 173.6 (166.6-180.5)  153.8 (138.8-168.7) | 0.969 | 1.00  1.03 (0.23-4.54) | 0.968 |
| **Chemotherapy**  No  Yes | 42  215 | 71.7  83.5 | 154.0 (132.5-175.5)  159.5 (149.5-169.4) | 0.515 | 1.00  0.82 (0.35-1.93) | 0.654 |  | 90.6  90.8 | 173.0 (157.8-188.2)  171.1 (164.1-178.0) | 0.919 | 1.00  0.94 (0.27-3.29) | 0.937 |
| **Therapy**  Neither RT nor CT  CT alone  CRT | 36  191  24 | 67.8  82.7  86.7 | 149.9 (126.1-173.7)  159.1 (148.4-169.9)  135.2 (116.4-154.0) | 0.375  0.535 | 1.00  0.66 (0.28-1.59)  0.72 (0.19-2.80) | 0.355  0.635 |  | 89.7  90.8  90.4 | 171.5 (154.7 - 188.2)  171.3 (163.8 - 178.8)  140.8 (124.8 - 156.9) | 0.741  0.956 | 1.00  0.80 (0.22-2.88)  1.03 (0.17-6.19) | 0.733  0.971 |
| **Stage IV** | | | | | | | | | | | | |
| **Radiotherapy**  No  Yes | 72  13 | 72.6  90.9 | 146.6 (129.5-163.6)  164.2 (136.5-191.9) | 0.308 | 1.00  0.45 (0.06-3.47) | 0.443 |  | 86.2  90.9 | 164.0 (150.4-177.6)  164.2 (136.5-191.9) | 0.758 | 1.00  1.13 (0.13-9.68) | 0.912 |
| **Chemotherapy**  No  Yes | 8  77 | 83.3  75.2 | 115.7 (90.0-141.3)  149.5 (133.6-165.4) | 0.810 | 1.00  1.86 (0.24-14.26) | 0.552 |  | 83.3  87.5 | 115.7 (90.0-141.3)  165.4 (152.7-178.2) | 0.766 | 1.00  1.01 (0.12-8.34) | 0.989 |
| **Therapy**  Neither RT nor CT  CT alone  CRT | 7  65  12 | 83.3  72.1  90.9 | 115.7 (90.0-141.3)  145.6 (127.6-163.6)  164.2 (136.5-191.9) | 0.740  0.751 | 1.00  1.42 (0.19-10.83)  0.51 (0.03-8.14) | 0.737  0.631 |  | 83.3  86.8  90.9 | 115.7 (90.0-141.3)  164.6 (150.5-178.8)  164.2 (136.5-191.9) | 0.796  0.751 | 1.00  0.76 (0.09-6.22)  0.57 (0.04-9.11) | 0.800  0.689 |
| Abbreviations: N, number of cases; OS, overall survival; CSS, cancer-specific survival; 10y OS, OS in 10 years; 10y CSS, CSS in 10 years; HR, hazard ratio; CI, confidence interval; NLPHL, Nodular lymphocyte predominant Hodgkin lymphoma; SEER, Surveillance, Epidemiology, and End Results; RT, radiotherapy; CT, chemotherapy; CRT, combined radiotherapy and chemotherapy. | | | | | | | | | | | | |
| ^1^Derived from Kaplan-Meier survival analysis. | | | | | | | | | | | | |
| ^2^Derived from multivariate Cox proportional -hazards models. Present here is the final model. Insignificant variables were dropped from the final multivariate analysis. | | | | | | | | | | | | |

| Supplementary table 2. Univariate and multivariate analysis of OS and CSS in stage I-IV NLPHL, respectively, after PSM, SEER 2000-2015. | | | | | | | | | | | | | |
| --- | --- | --- | --- | --- | --- | --- | --- | --- | --- | --- | --- | --- | --- |
| Therapy | N | OS(months) | | | | | |  | CSS(months) | | | | |
|  |  | 10y OS (%) | | Mean (95% CI) | *P*^1^ | HR(95% CI) | *P^2^* |  | 10y CSS (%) | Mean (95% CI) | *P*^1^ | HR(95% CI) | *P^2^* |
| **All stage** | | | | | | | | | | | | | |
| **^3^PSM model 1**  Neither RT nor CT (as ref.)  CT alone  CRT | 218  206  201 | 85.8  91.1  96.6 | 168.0 (160.2-175.8)  173.6 (167.0-180.1)  182.2 (177.3-187.1) | | 0.376  0.011 | 1.00  0.58 (0.28-1.17)  0.42 (0.18-0.99) | 0.128  0.047 |  | 95.4  93.9  98.3 | 178.9 (173.8-184.1)  177.3 (171.8-182.9)  186.3 (183.2-189.3) | 0.857  0.092 | 1.00  1.09 (0.43-2.75)  0.34 (0.09-1.26) | 0.855  0.107 |
| **^4^PSM model 2**  Neither RT nor CT (as ref.)  RT alone  CRT | 184  156  225 | 84.7  92.5  96.9 | 166.9 (158.3-175.6)  180.2 (173.2-187.1)  183.1 (178.9-187.4) | | 0.092  0.009 | 1.00  0.31 (0.12-0.80)  0.38 (0.16-0.92) | 0.017  0.033 |  | 94.1  99.4  98.5 | 178.5 (172.7-184.3)  187.8 (185.5-190.1)  186.5 (183.8-189.3) | 0.052  0.066 | 1.00  0.12 (0.02-1.00)  0.41 (0.11-1.57) | 0.049  0.193 |
| **Stage I** | | | | | | | | | | | | | |
| **^3^PSM model 1**  Neither RT nor CT (as ref.)  CT alone  CRT | 101  100  91 | 88.3  97.9  98.4 | 166.9 (156.2-177.5)  182.3 (177.3-187.4)  187.1 (183.5-190.8) | | 0.052  0.013 | 1.00  0.21 (0.04-0.99)  0.15 (0.02-1.26) | 0.049  0.081 |  | 94.8  97.9  100.0 | 166.9 (156.2-177.5)  182.3 (177.3-187.4)  / | 0.052  / | 1.00  0.46 (0.08-2.51)  / | 0.368 |
| **^4^PSM model 2**  Neither RT nor CT (as ref.)  RT alone | 110  107 | 87.9  96.6 | 166.3 (156.4-176.2)  183.7 (177.7-189.6) | | 0.040 | 1.00  0.12 (0.02-0.64) | 0.013 |  | 95.3  97.8 | 176.3 (169.9-182.8)  185.5 (180.6-190.4) | 0.227 | 1.00  0.25 (0.03-2.25) | 0.216 |
| **Stage II** | | | | | | | | | | | | | |
| **^3^PSM model 1**  Neither RT nor CT (as ref.)  CT alone  CRT | 33  39  69 | 89.3  80.0  98.5 | 168.2 (150.1-186.3)  146.7 (129.0-164.4)  175.0 (170.2-179.9) | | 0.486  0.371 | 1.00  1.62 (0.27-9.71)  0.40 (0.06-2.86) | 0.596  0.362 |  | 93.8  91.6  100.0 | 168.2 (150.1-186.3)  146.7 (129.0-164.4)  / | 0.486  / | 1.00  1.38 (0.26-7.45)  / | 0.709  / |
| **Stage III** | | | | | | | | | | | | | |
| **^3^PSM model 1**  Neither RT nor CT (as ref.)  CT alone | 36  30 | 67.8  95.7 | 149.9 (126.1-173.7)  111.4 (104.5-118.3) | | 0.171 | 1.00  0.25 (0.03-2.12) | 0.204 |  | 100.0  100.0 | /  / | 0.140 | -  - | - |
| **Stage IV** | | | | | | | | | | | | | |
| **^3^PSM model 1**  Neither RT nor CT (as ref.)  CT alone | 7  8 | 83.3  75.0 | 115.7 (90.0-141.3)  96.3 (61.7-130.8) | | 0.468 | 1.00  2.36 (0.21-27.2) | 0.491 |  | 83.3  87.5 | 115.7 (90.0-141.3)  110.6 (84.3-137.0) | 0.846 | 1.00  1.32 (0.08-22.44) | 0.846 |
| Abbreviations: N, number of cases; PSM, propensity score matching; OS, overall survival; CSS, cancer-specific survival; 10y OS, OS rate at 10 years; 10y CSS, CSS rate at 10 years; HR, hazard ratio; CI, confidence interval; NLPHL, Nodular lymphocyte predominant Hodgkin lymphoma; SEER, Surveillance, Epidemiology, and End Results; RT, radiotherapy; CT, chemotherapy; CRT, combined radiotherapy and chemotherapy. | | | | | | | | | | | | | |
| ^1^Derived from Kaplan-Meier survival analysis. | | | | | | | | | | | | | |
| ^2^Derived from multivariate Cox proportional -hazards models. | | | | | | | | | | | | | |
| ^3^PSM model 1: PSM was performed to match patients treated with chemotherapy and those treated without chemotherapy.  ^4^PSM model 2: PSM was performed to match patients treated with radiotherapy and those treated without radiotherapy.  Different covariables were applied for PSM in different stages. | | | | | | | | | | | | | |
| ^5^ “/”means no patient reached an end event. | | | | | | | | | | | | | |
| ^6^“-” means coefficient does not converge. | | | | | | | | | | | | | |

| Supplementary table 3. Univariate and multivariate analysis of OS and CSS in advanced stage NLPHL, before and after PSM, SEER 2000-2015. | | | | | | | | | | | | |
| --- | --- | --- | --- | --- | --- | --- | --- | --- | --- | --- | --- | --- |
| Therapy | N | OS (months) | | | | |  | CSS (months) | | | | |
|  |  | 10y OS (%) | Mean (95% CI) | *P*^1^ | HR (95% CI) | *P^2^* |  | 10y CSS (%) | Mean (95% CI) | *P*^1^ | HR (95% CI) | *P^2^* |
| **Before PSM** | | | | | | | | | | | | |
| Neither RT nor CT (as ref.)  CT alone  RT alone  CRT | 43  256  7  36 | 69.2  79.7  ^3^/  88.3 | 151.1 (129.3-172.9)  156.3 (147.1-165.5)  ^3^/  160.2 (142.8-177.5) | 0.621  ^3^/  0.396 | 1.00  0.87 (0.40-1.89)  ^3^/  - | 0.716  ^3^/  - |  | 88.2  89.9  ^3^/  90.8 | 169.6 (153.4-185.7)  170.3 (163.7-176.9)  ^3^/  164.6 (149.0-180.1) | 0.772  ^3^/  0.915 | 1.00  1.00 (0.34-2.98)  ^3^/  - | 0.998  ^3^/  - |
| **After PSM** | | | | | | | | | | | | |
| **^4^****PSM model 1**  **CT**  No (as ref.)  Yes | 50  50 | 87.8  94.6 | 154.5 (134.5-174.4)  160.1 (142.1-178.2) | 0.303 | 1.00  0.39 (0.11-1.33) | 0.133 |  | 92.7  97.4 | 171.0 (156.1-185.9)  176.1 (168.4-183.7) | 0.182 | 1.00  0.25 (0.03-2.25) | 0.217 |
| **^5^PSM model 2**  **RT**  No (as ref.)  Yes | 43  43 | 89.6  89.7 | 152.0 (131.0-172.9)  162.5 (147.2-177.9) | 0.310 | -  - | - |  | 92.3  91.8 | 170.1 (154.4-185.8)  166.2 (152.4-180.1) | 0.826 | 1.00  1.70 (0.33-8.84) | 0.526 |
| **^4^PSM model 1**  Neither RT nor CT (as ref.)  CT alone | 43  45 | 69.2  75.9 | 151.1 (129.3-172.9)  157.0 (136.4-177.6) | 0.318 | 1.00  0.40 (0.12-1.36) | 0.143 |  | 88.2  97.1 | 169.6 (153.4-185.7)  175.6 (167.1-184.1) | 0.190 | 1.00  0.26 (0.03-2.30) | 0.224 |
| **^5^PSM model 2**  Neither RT nor CT (as ref.)  CRT | 37  36 | 67.2  88.3 | 149.9 (126.2-173.7)  160.2 (142.8-177.5) | 0.393 | -  - | -  - |  | 86.3  90.8 | 166.8 (148.2-185.4)  164.6 (149.0-180.1) | 0.766 | -  - | -  - |
| **Abbreviations**: N, number of cases; OS, overall survival; CSS, cancer-specific survival; 10y OS, OS in 10 years; 10y CSS, CSS in 10 years; HR, hazard ratio; CI, confidence interval; NLPHL, Nodular lymphocyte predominant Hodgkin lymphoma; SEER, Surveillance, Epidemiology, and End Results; RT, radiotherapy; CT, chemotherapy; CRT, combined radiotherapy and chemotherapy; PSM, propensity score matching. | | | | | | | | | | | | |
| ^1^Derived from Kaplan-Meier survival analysis. | | | | | | | | | | | | |
| ^2^Derived from final multivariate Cox proportional -hazards models. | | | | | | | | | | | | |
| ^3^ “/” means no patient reached an end event. | | | | | | | | | | | | |
| ^4^PSM model 1: PSM was performed to match patients treated with chemotherapy and those treated without chemotherapy.  ^5^PSM model 2: PSM was performed to match patients treated with radiotherapy and those treated without radiotherapy. | | | | | | | | | | | | |
| ^6^ “-” means PH was not meet. | | | | | | | | | | | | |

| Supplementary table 4. Univariate and multivariate analysis of OS and CSS in stage I-IV NLPHL after PSM, SEER 1983-2015. | | | | | | | | | | | |
| --- | --- | --- | --- | --- | --- | --- | --- | --- | --- | --- | --- |
| Therapy | N | OS(months) | | | | |  | CSS(months) | | | |
|  |  | Mean (95% CI) | *P*^1^ | HR(95% CI) | | *P^2^* |  | Mean (95% CI) | *P*^1^ | HR(95% CI) | *P^2^* |
|  | | **1983-2015** | | | | | | | | | |
|  |  | **Early stage** | | | | | | | | | |
| Neither RT nor CT (as ref.)  CT alone  RT alone  CRT | 162  202  162  202 | 220.0 (201.5-238.6)  296.5 (269.1-323.9)  279.0 (261.5-296.5)  261.1 (248.9-273.2) | 0.089  0.035  0.001 | 1.00  0.61 (0.31-1.17)  0.48 (0.23-1.01)  0.31 (0.12-0.79) | | 0.133  0.053  0.014 |  | 244.9 (233.4-256.4)  320.0 (297.8-342.2)  299.5 (289.2-309.8)  268.2 (259.9-276.6) | 0.163  0.066  0.010 | 1.00  0.54 (0.21-1.36)  0.34 (0.11-1.10)  0.27 (0.07-0.99) | 0.189  0.071  0.047 |
|  |  | **Advanced stage** | | | | | | | | | |
| Neither RT nor CT (as ref.)  Either CT or RT  CRT | 42  13  37 | 187.2 (144.4-230.1)  232.0 (160.3-303.7)  243.1 (216.4-269.8) | 0.709  0.201 | 1.00  0.72 (0.20-2.66)  1.13 (0.33-3.89) | 0.626  0.847 | |  | 242.2 (210.0-274.4)  282.0 (231.5-332.5)  249.7 (225.6-273.8) | 0.795  0.762 | 1.00  0.72 (0.08-6.23)  1.40 (0.29-6.72) | 0.765  0.671 |
|  | | **1983-2000** | | | | | | | | | |
|  |  | **Early stage** | | | | | | | | | |
| Neither RT nor CT (as ref.)  CT alone  RT alone  CRT | 22  22  22  22 | 204.0 (168.4-239.6)  278.5 (228.6-328.4)  266.3 (233.0-299.6)  243.8 (213.8-273.8) | 0.390  0.272  0.201 | 1.00  0.61 (0.20-1.86)  0.55 (0.18-1.68)  0.46 (0.14-1.54) | 0.382  0.293  0.210 | |  | 225.9 (191.9-259.9)  302.8 (260.9-344.8)  300.7 (285.0-316.5)  248.5 (219.1-277.9) | 0.718  0.147  0.644 | 1.00  0.74 (0.17-3.29)  0.23 (0.03-2.01)  0.72 (0.16-3.22) | 0.688  0.182  0.666 |
|  |  | **Advanced stage** | | | | | | | | | |
| Neither RT nor CT (as ref.)  Either CT or RT alone | 9  17 | 195.7 (136.2-255.2)  242.9 (175.9-309.9) | 0.969 | 1.00  0.98 (0.28-3.35) | 0.969 | |  | 237.4 (182.9-291.9)  299.5 (239.3-359.7) | 0.894 | 1.00  0.89 (0.15-5.31) | 0.894 |
| Abbreviations: N, number of cases; PSM, propensity score matching; OS, overall survival; CSS, cancer-specific survival; 10y OS, OS in 10 years; 10y CSS, CSS in 10 years; HR, hazard ratio; CI, confidence interval; NLPHL, Nodular lymphocyte predominant Hodgkin lymphoma; SEER, Surveillance, Epidemiology, and End Results; RT, radiotherapy; CT, chemotherapy; CRT, combined radiotherapy and chemotherapy. | | | | | | | | | | | |
| ^1^Derived from Kaplan-Meier survival analysis. | | | | | | | | | | | |
| ^2^Derived from multivariate Cox proportional-hazards models. | | | | | | | | | | | |
| Different covariables were applied for PSM at different stages. | | | | | | | | | | | |
